# NLRP3 inflammasome-induced pyroptosis in digestive system tumors

**DOI:** 10.3389/fimmu.2023.1074606

**Published:** 2023-04-04

**Authors:** Jiexia Wen, Bin Xuan, Yang Liu, Liwei Wang, Li He, Xiangcai Meng, Tao Zhou, Yimin Wang

**Affiliations:** ^1^Department of Central Laboratory, The First Hospital of Qinhuangdao, Hebei Medical University, Qinhuangdao, Hebei, China; ^2^Department of General Surgery, The First Hospital of Qinhuangdao, Hebei Medical University, Qinhuangdao, Hebei, China

**Keywords:** NLRP3, inflammasome, digestive system tumors, pyroptosis, cancer

## Abstract

Programmed cell death (PCD) refers to cell death in a manner that depends on specific genes encoding signals or activities. PCD includes apoptosis, pyroptosis, autophagy and necrosis (programmed necrosis). Among these mechanisms, pyroptosis is mediated by the gasdermin family and is accompanied by inflammatory and immune responses. When pathogens or other danger signals are detected, cytokine action and inflammasomes (cytoplasmic multiprotein complexes) lead to pyroptosis. The relationship between pyroptosis and cancer is complex and the effect of pyroptosis on cancer varies in different tissue and genetic backgrounds. On the one hand, pyroptosis can inhibit tumorigenesis and progression; on the other hand, pyroptosis, as a pro-inflammatory death, can promote tumor growth by creating a microenvironment suitable for tumor cell growth. Indeed, the NLRP3 inflammasome is known to mediate pyroptosis in digestive system tumors, such as gastric cancer, pancreatic ductal adenocarcinoma, gallbladder cancer, oral squamous cell carcinoma, esophageal squamous cell carcinoma, in which a pyroptosis-induced cellular inflammatory response inhibits tumor development. The same process occurs in hepatocellular carcinoma and some colorectal cancers. The current review summarizes mechanisms and pathways of pyroptosis, outlining the involvement of NLRP3 inflammasome-mediated pyroptosis in digestive system tumors.

## 1 Introduction

Cancer has become a major cause of mortality in an aging world population, necessitating clinical and basic research to aid treatment and survival rates. The programmed cell death (PCD) model of pyroptosis leads to a release of intracellular pro-inflammatory mediators, causing inflammation and promoting tumor progression, a process known to involve the NLRP3 inflammasome. The four pathways of pyroptosis and the role of NLRP3 inflammasome-mediated pyroptosis in digestive system tumors are reviewed below with the intention of aiding progress in the treatment of digestive tract-related tumors. Cells may die as a result of necrosis or PCD (1–8). PCD refers to spontaneous cell death in response to the activation of specific genes and includes apoptosis, pyroptosis, autophagy and necroptosis (1, 9). Pyroptosis involves the formation of cell membrane pores mediated by gasdermin proteins causing ion transport, accompanied by inflammation and an immune response (1). Ion imbalance leads to cell swelling, lysis and release of pro-inflammatory factors, including interleukin (IL)-1β, IL-18, adenosine triphosphate (ATP) and high mobility group box 1 (HMGB1) protein (10). Pyroptosis is considered to have ambiguous roles in tumorigenesis, inhibiting the growth of some tumors and stimulating a pro-inflammatory microenvironment that promotes the growth of other tumor-types (1, 11–13). The process has attracted attention for its potential as regards anti-tumor therapy. Four pyroptosis-inducing pathways have been described: the classical caspase-1-dependent, the non-canonical caspase-4/5/11-dependent, the apoptosis-pyroptosis transition involving high expression of Gasdermin-E (GSDME) and the granzyme-induced pathways (1, 9, 10). Chemotherapeutic drugs which activate caspase-3 to cleave GSDME, induce a switch from apoptosis to pyroptosis which causes cell death (14). Moreover, granzyme B induces pyroptosis *via* GSDME cleavage (15) and granzyme A activates GSDMB pore-forming and pyroptosis (16). The most studied remains the classical pyroptosis pathway (1), involving an initiation and an activation signal (17), in which the inflammasome plays a key role.

## 2 Overview of NLRP3 inflammasome

### 2.1 Composition and properties of the NLRP3 inflammasome

**Figure 1:**
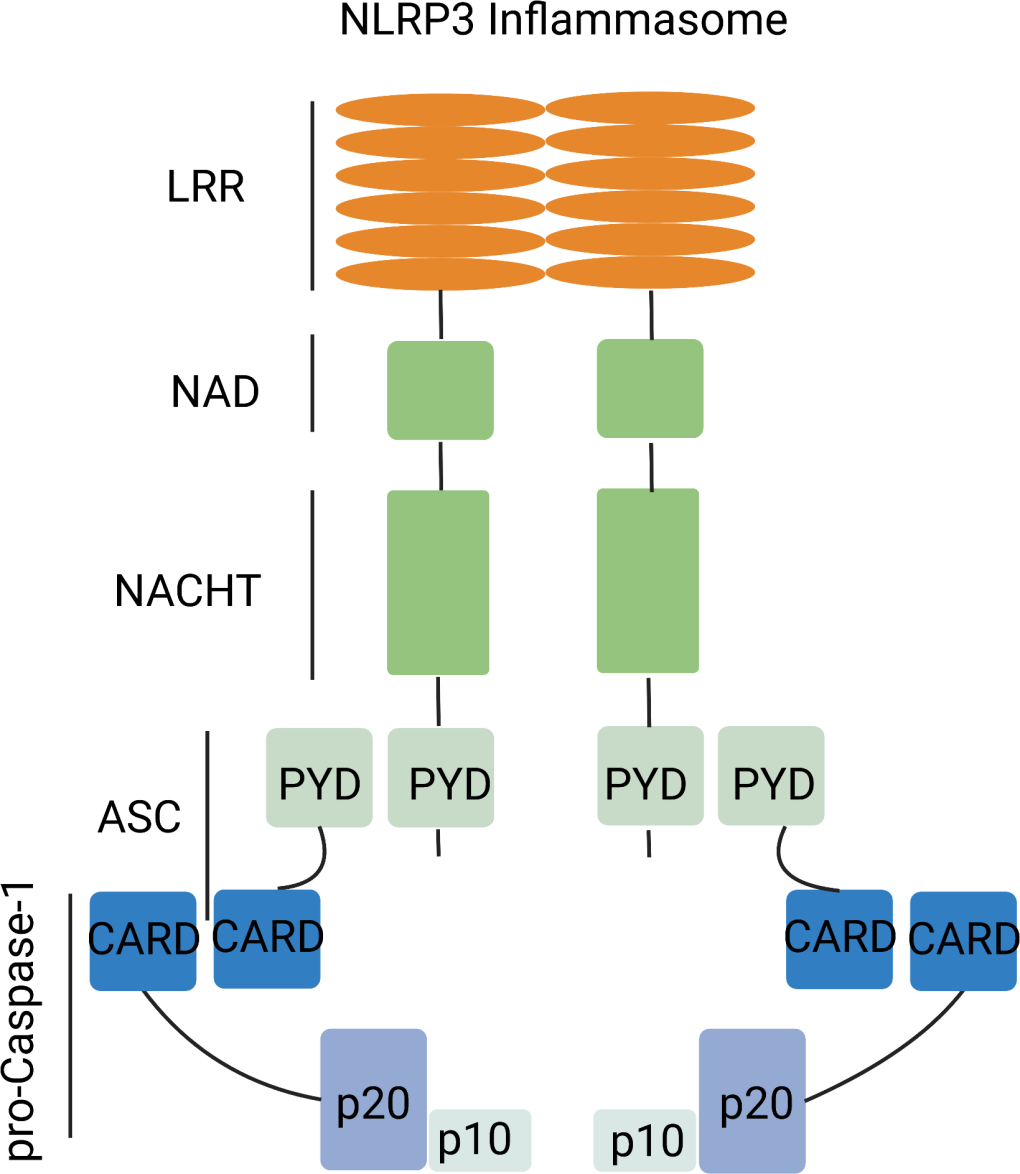
NLRP3 inflammasome complex. The NLRP3 inflammasome complex has a central NACHT domain flanked by a C-terminal leucine-rich repeat (LRR) and an N-terminal pyrin domain (PYD). NLRP3 activation allows interaction with ASC. ASC further interacts with pro-caspase-1.

The cytosolic inflammasome is a multiprotein signaling complex, comprising pattern recognition receptor (PRR), apoptosis-associated speck-like protein containing a CARD (ASC) and pro-Caspase-1 (18). PRRs differ by subcellular localization (19) with Toll-like receptor (TLR) and C-type lectin (CLR), which recognize extracellular damage-associated molecular patterns (DAMPs) and pathogen-associated molecular patterns (PAMPs) that are located in the plasma membrane and endosome. By contrast, RIG-I-like receptor (RLR), absent in melanoma 2 (AIM2), AIM2-like receptor (ALR), nucleotide- binding and oligomerization (NOD), NOD-domain like receptor (NLR) and cytosolic sensor cyclic GMP-AMP (cGAMP) synthase (cGAS) influence intracellular compartmentalization, including retinoic acid-inducible genes (19, 20). The much-studied NLRP3 inflammasome participates in the innate immune system (21) and is expressed by antigen presenting cells (APC) and inflammation-activated cells, including macrophages, dendritic cells (dendritic cells, DC), neutrophils and monocytes (22). The NLRP3 inflammasome consists of a sensor (NLRP3), an adaptor (ASC) and an effector (caspase-1). NLRP3 is a tripartite protein that contains an amino-terminal pyrin domain (PYD), a central NACHT domain and a carboxy-terminal leucine-rich repeat (LRR) domain. The NACHT domain has ATPase activity that is vital for NLRP3 self-association and function (23), whereas the LRR domain is thought to induce autoinhibition by folding back onto the NACHT domain. The adaptor ASC has two protein interaction domains, an N-terminal PYD and a C-terminal caspase-recruitment domain (CARD). Full-length caspase-1 has an N-terminal CARD, a central large catalytic domain (p20) and a C-terminal small catalytic subunit domain (p10). Upon stimulation, NLRP3 oligomerizes through homotypic interactions between NACHT domains (**Figure 1**) (24, 25).

### 2.2 Activation and regulation of the NLRP3 inflammasome

**Figure 2:**
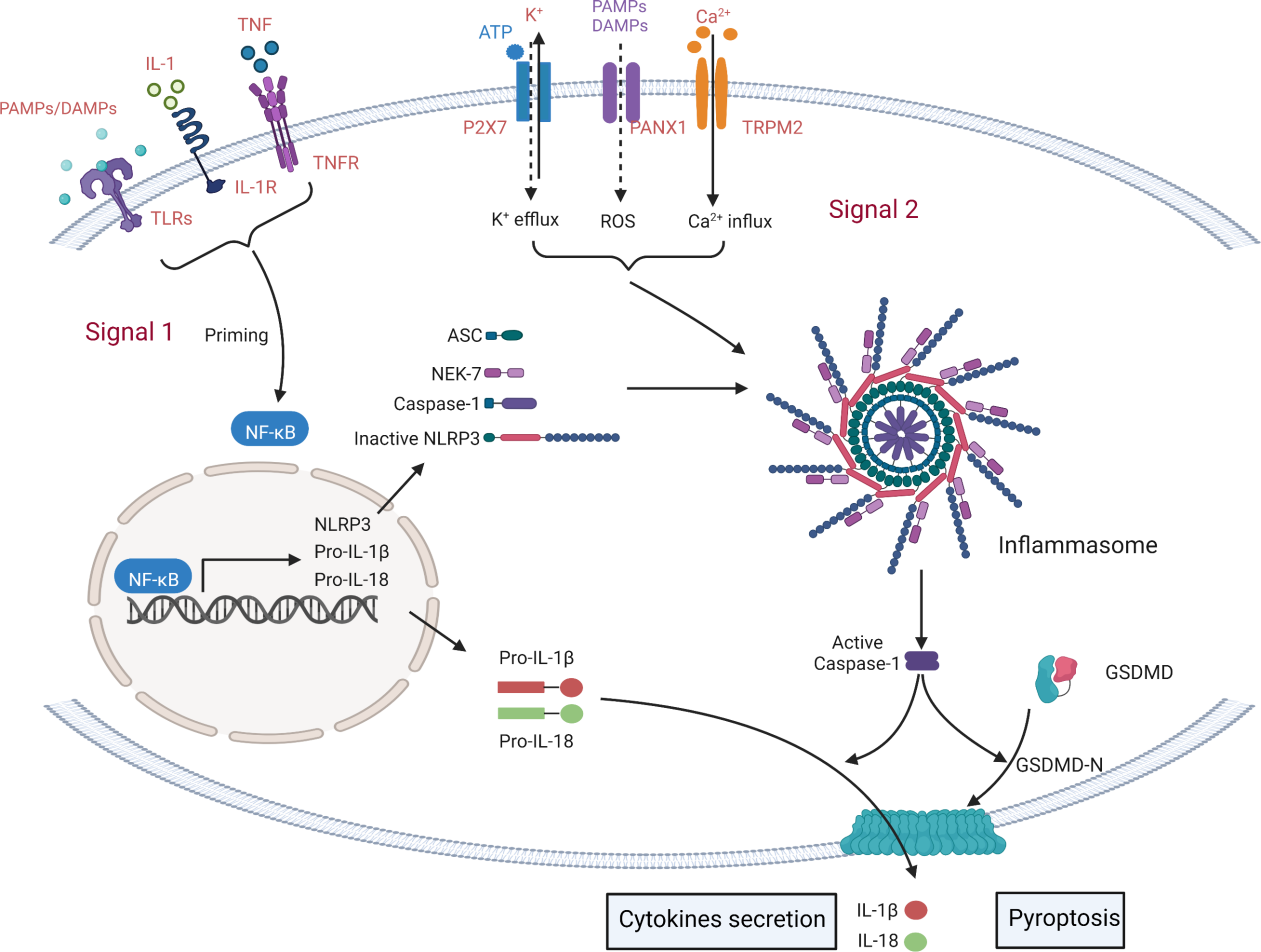
Simplified mechanisms for the canonical NLRP3- inflammasome activation. The formation of inflammasome requires two key steps: NLRP3 initiation signal and activation signal. The initiation signal is triggered by Toll-like receptors (TLRs) and cytokine recognition receptors that recognize PAMPs/DAMPs and cytokines (including IL1, TNF). These signals induce the transcription of NLRP3, pro-IL-1β, pro-IL-18 and pro-Caspase1 through NF-κB. Secondary signals are triggered by a wide range of stimuli, including K^+^ efflux, Ca^2+^ influx, phagocytosis of microbial and particulate matter (leading to destabilization/rupture and release of lysosomal cathepsins and reactive oxygen species (ROS)), and Mitochondrial dysfunction. Then, a multi-protein complex composed of NOD-like receptor protein, ASC and pro-caspase-1 is activated to activate the NLRP3 inflammasome, triggering the release of IL-1β and IL-18 and cell pyroptosis.

NLRP3 is activated by a sequence of two signals (**Figure 2**). 1) Inflammatory stimuli produced by TLR ligands or endogenous molecules induce NF-κB expression (19) and reactive oxygen species (ROS), hypoxia, metabolites, oxidized low density lipoprotein (oxLDL), amyloid and complement may all be activated during non-pathogen responsive sterile inflammatory diseases (24, 26). 2) PAMPs and DAMPs trigger potassium (K^+^) efflux, increased calcium (Ca^2+^) flux, lysosomal damage or ROS production (26). The activated NLRP3 undergoes a conformational change to expose the NACHT domain and promote oligomerization (19) which allows the binding of the PYD domain to ASC (PYCARD) (17). Pro-caspase-1 is recruited and cleaved by the NLRP3-ASC complex in a CARD-CARD homotypic interaction, resulting in the activated NLRP3 inflammasome, consisting of a NLRP3-ASC-Caspase-1 complex (19). The NLRP3 inflammasome then mediates pyroptosis by cleaving gasdermin proteins (6), including GSDMA, GSDMB, GSDMC, GSDMD and GSDME/DFNA5 (18), which differ in their mechanisms of pyroptosis induction. GSDMD is the main substrate of NLRP3 inflammasome-induced pyroptosis, although GSDME regulates the granzyme pathway or Caspase-3 (6). Other gasdermins have been linked to pyroptosis but little is known about NLRP3 interactions. GSDMD has an N-terminal pore-forming domain and a C-terminal auto-inhibitory domain (27). On activation by caspase-1, cleavage separates the N- and C-terminal domains disabling auto-inhibition and activating pore-formation (28, 29). Caspase-1 also cleaves and activates pro-IL-1β and pro-IL-18 which may be released through the GSDMD-N-terminal domain channel, triggering an inflammatory response (29).

### 2.3 NLRP3 activation in the tumor microenvironment

TME is closely related to tumorigenesis, and inflammation and persistent infection may lead to various human malignancies. Studies have demonstrated that NLRP3 inflammasome polymorphisms are associated with different malignancies such as colon cancer and melanoma (30). NLRP3 inflammasome can be activated by microbial cell wall components and toxins (31). In addition, NLRP3 inflammasomes are also proficient in sensing stress or endogenous danger signals, including extracellular ATP (32), extracellular glucose (33), crystalloids, reactive oxidants (ROS) and amyloid β fibrils (34). Cellular phagocytosis of amyloid β fibrils causes destabilization of the lysosome, causing release of contents (histone protease) and ROS, which initiates activation of NLRP3 (35). Recent studies have found that β2m accumulated in multiple myeloma (MM) is taken up by macrophages, leading to the aggregation of β-fibers in lysosomes, causing lysosomal rupture and activation of the NLRP3 inflammasome (36).

## 3 NLRP3 inflammasome and digestive system tumors

Extracellular inflammatory responses induced by pyroptosis lead to the removal of dead or damaged cells but may also aggravate the pre-existing disease. Both of these effects influence the impact of NLRP3 inflammasome-induced pyroptosis on tumors.

### 3.1 Oral squamous cell carcinoma

Oral squamous cell carcinoma (OSCC) accounts for over 90% of oral cancers (37) and has a poor prognosis (38, 39). Tumor-associated chronic inflammation has been described as the 7th biological feature of malignancy (40). Chronic inflammation influences tumorigenesis, development, migration and invasion through the NF-κB-IL-6-STAT pathway but also recruits immune and inflammatory cells into tumor tissues. With tumor progression, immune and inflammatory cells switch from immune surveillance and tumor suppression to tumor promotion (41). Abnormal activation of the NLRP3 inflammasome has been associated with various chronic inflammation (19) and is known to be overexpressed in OSCC cells and tissues, allowing it to be associated with tumor stage and lymph node metastasis (42, 43). Indeed, NLRP3 knockdown inhibited OSCC proliferation, migration and invasion (44). IL-6 is a multifunctional immune and inflammatory molecule (45) which is overexpressed in cancer. IL-6 activates NLRP3, causing secretion of IL-1β/IL-18 and promoting OSCC cell proliferation, migration and invasion. Indeed, NLRP3 silencing prevented the IL-6-mediated proliferation and NLRP3 inflammasome activation in OSCC cells. In addition, the NLRP3 pathway can participate in the IL-6-mediated OSCC process as a downstream target of Sox4 (46).

### 3.2 Esophageal squamous cell carcinoma

Esophageal cancer (EC) has a high incidence with distinct geographical demarcation (47) which makes esophageal squamous cell carcinoma (ESCC) the most common pathological form of EC in China and other parts of Asia (48). Epidemiological studies have linked the environmental carcinogens, nitrosamines, to EC and gastric cancer (49, 50). Nitrosamines trigger an inflammatory response and participate in the malignant transformation of cells. Nitrosamine exposure of esophageal epithelial Het-1A cells caused ROS-production to trigger pyroptosis and the inflammatory response *via* the NLRP3/caspase-1/GSDMD canonical pathway (51). NLRP3 inflammasome levels have been shown to be higher in ESCC tumor tissues than in noncancerous tissues and to correlate positively with the Ki-67 proliferation index (52). NLRP3 protein expression was also shown to correlate with tumor node metastasis (TNM) and T stage but not with lymph node or metastasis status, gender or age. Patients with higher NLRP3 expression were suggested to have a more malignant clinical phenotype. In addition, knockdown or overexpression of NLRP3 in ESCC cell lines had the respective effects of abrogation or promotion of cell migration and invasion. Thus, NLRP3 inflammasome activity appears to contribute to ESCC development and progression (52).

### 3.3 Gastric cancer

Gastric cancer is a high-incidence malignant tumor (53) related to factors such as Helicobacter pylori, smoking and drinking. Chronic inflammation may be a cause of gastric cancer (54), through microenvironmental and metabolic changes which promote proliferation, invasion and migration of tumor cells (55). Helicobacter pylori (Hp) has been implicated in many digestive system diseases, such as chronic and atrophic gastritis and gastric ulcer (56), and is a susceptibility factor for gastric cancer (57, 58). HP infection is considered the strongest single risk factor for gastric cancer and NLRP3 may be involved *via* the production of IL-1β (59). The pro-inflammatory IL-1β has been shown to be involved in gastric mucosal atrophy, intestinal metaplasia, dysplasia and other pathological changes of gastric mucosa (60) and induces gastric mucosal atrophy by interfering with the Sonic hedgehog (SHH) pathway in parietal cells (61). Rats with the high gastric mucosal expression of IL-1β had a greater incidence of gastric mucosal dysplasia and inflammatory cell infiltration, even in the absence of HP infection than those with low expression (62). In HP-induced gastric cancer, NOD1 protein and its inflammatory effects were significantly increased (63). The NLRP3 inflammasome regulates cyclin-D1, inducing IL-1β production to enhance differentiation of gastric cancer cells. The IL-1β-dependent activation of NF-κB stimulated the c-Jun N-terminal kinase (JNK) signaling pathway, leading to tumor proliferation, invasion and progression (11). Thus, the NLRP3 inflammasome promotes gastric cancer and its downregulation by the activity of the aryl hydrocarbon receptor (AhR), dopamine receptor D1 (DRD1) and G protein-coupled bile acid receptor 1, (GPBAR1) may limit the occurrence of pyroptosis, thereby affecting cancer progression (19).

### 3.4 Liver cancer

Primary liver cancer (PLC) refers to cancer that occurs in hepatocytes or intrahepatic cholangiocarcinoma, including hepatocellular carcinoma (HCC), intrahepatic cholangiocarcinoma (ICC), and mixed types of both (64, 65). Usually, HCC develops through three processes, hepatitis, cirrhosis and primary liver cancer (66–70). Pyroptosis can be involved in precancerous and malignant development of primary liver cancer. Hepatic fibrosis and cirrhosis are the initiating factors for the development of HCC, and to some extent, cirrhosis is the precancerous stage of PLC. Liver cells are mainly composed of hepatocytes, hepatic stellate cells (HSC), bile duct epithelial cells, natural killer cells, and Kupffer cells (71). HSC are the main type of hepatic fibrotic cells, and HSC proliferation and activation are the key steps in liver fibrosis. Research shows that when HSC are stimulated by mediators released from blast cells or inflammatory cells, inflammasome induce pyroptosis by activating caspase-1 and releasing pro-inflammatory factors IL-1β and IL-18, which in turn drive the progression of liver fibrosis (72). NLRP3 inflammasome may play a direct role in HSC activation and liver fibrosis. In NLRP3 overexpressing HCC mice, it was found that the mice had shorter survival time, poor growth, neutrophil infiltration and hepatic stellate cell activation, severe hepatitis response, and significant liver fibrosis (73). *In vitro* experiments have shown that extracellular ATP can activate HSC NLRP3 *via* the purinoceptor P2X ligand-gated cation channel 7 (P2X7) and promote the release of fibrotic markers such as α-SMA and type I collagen leading to liver fibrosis. P2X7R-mediated NLRP3 activation involved in IL-1β production by hepatic stellate cells may be associated with extracellular matrix deposition, suggesting that blocking the P2X7R-NLRP3 axis may be a potential therapeutic target for liver fibrosis (74). Transforming growth factor-β1 (TGF-β1) is a key mediator of tissue fibrosis and dysregulation of the TGF-β1 pathway is an important pathogenic mechanism of liver fibrosis (75). Activation of HSC leads to TGF-β1 production, which in turn binds to transforming growth factor β (TGFβ), leading to TGFβ pathway activation and HSC activation, thus promoting the process of liver fibrosis (76, 77). Aldosterone can induce HSC activation and liver fibrosis in mice by promoting NLRP3 assembly and expression (78). The above studies suggest that pyroptosis-mediated inflammation can induce HSC activation and promote liver fibrosis.

In addition, it has been shown that angiotensin Ang II induces caspase-1-mediated hepatocyte pyroptosis by upregulating the levels of reactive oxygen species and NOX4 protein in hepatocytes and promoting the expression of NLRP3 inflammasome secretion axis-related proteins (NLRP3, ASC, Caspase-1, IL-1β). This suggests that hepatocyte activation by NLRP3 leads to a significant increase in cysteinase activity, which in turn induces hepatocyte pyroptosis. Therefore, caspase inhibitors can be used to inhibit hepatocyte pyroptosis to suppress the progression of liver fibrosis (73, 79). In addition to inflammasome, IL-1β and gasdermin proteins are also important molecules that cause cirrhosis and liver fibrosis. IL-1β can induce the conversion of microvascular endothelial cells into myofibroblasts, leading to the proliferation of collagenous tissue. It can also directly activate hepatic stellate cells, promote the expression of inflammatory factors such as TNF-α and stimulate the inflammatory cascade response, gradually developing liver fibrosis and even cirrhosis. TGF-β1 is one of the important pathways that contribute to liver fibrosis (75). It was found that TGF-β1 can inhibit caspase-1 expression and suppress IL-1β release, but this pathway has no significant effect on IL-18. The above data suggest that during the progression of PLC, pyroptosis induces fibrosis in the liver tissue, which leads to the development of PLC.

The function of pyroptosis is different in different stages of cancer development and progression. In the precancerous stage of liver fibrosis and cirrhosis, the accumulation of inflammasome and inflammatory factors will intensify the transformation of cirrhosis to PLC; while in the tumor stage, when cancer cells are formed, pyroptosis is inhibited, forming an intrinsic malignant microenvironment that blocks cancer cell death and accelerates the progression of PLC to the malignant level. The expression of estrogen receptor β (ERβ) and NLRP3 were reported to be significantly downregulated in liver tissues of patients with HCC, and their expression levels were positively correlated; estrogen can inhibit HCC cell proliferation and metastasis by activating NLRP3 through the ERβ/mitogen-activated protein kinase (MAPK) pathway (80, 81). Lin et al. (82) found that the levels of interferon-inducible nucleoprotein 16 (IFI16) were lower in HCC tissues than in normal tissues. Overexpression of IFI16 reduced cell viability, which led to significant inhibition of tumor growth and reduction of tumor size in HCC cells. Meanwhile, overexpression of IFI16 could activate inflammasome through caspase-1 and thus increase the levels of IL-1β and IL-18. Caspase-1 inhibitor (Ac-YVAD-CMK) could effectively inhibit the tumor suppressive effect of IFI16, thus it can be speculated that the tumor suppressive effect of IFI16 may be closely related to caspase-1-mediated pyroptosis. FUN14 structural domain protein 1 (FUNDC1) is a characteristic mitogenic receptor in most human HCC, and knockdown of FUNDC1 activates NLRP3 inflammasome to promote hepatocarcinogenesis during diethylnitrosamine (DEN)-induced hepatocarcinoma in mice (83). High mobility group protein 1 (HMGB1), a nuclear damage-associated molecule released under hypoxic stress, activates caspase-1 to promote HCC cell invasion and metastasis (84). Hepatitis C virus (HCV) also affects HCC scorching through its effect on NLRP3 inflammasome (85). In conclusion, the activation of NLRP3, a key molecule in pyroptosis, is closely related to the pathogenesis of HCC, and may provide a new strategy for HCC treatment by regulating pyroptosis.

### 3.5 Gallbladder cancer

Gallbladder cancer (GBC) is a highly malignant tumor, usually an adenocarcinoma, of the biliary system with a median survival time of only 6 months (86–88). Golgi phosphoprotein 3 (GOLPH3) has been shown to promote tumor progression in a variety of gastrointestinal malignancies. GOLPH3 and NLRP3 were shown to be highly upregulated in clinical GBC samples and levels of each were positively correlated with one another and with Ki-67 expression (89). GOLPH3 may be the upstream factor of NLRP3. Excessive activation of GOLPH3 leads to Golgi fragmentation, which is closely related to NLRP3 activation (85, 90, 91). In addition, PtdIns4p is required when NLRP3 is activated, of which, the free amount of PtdIns4p is associated with GOLPH3 (85, 92, 93). Moreover, there is evidence that mTOR can affect the activation of NLRP3 inflammasomes by regulating reactive oxygen species, and the activation of mTOR is also largely regulated by GOLPH3 (94, 95). Additionally, GOLPH3 and NLRP3 have been reported to both be regulated by the same upstream protein PD2 (95, 96). Further studies confirmed that GOLPH3 enhanced GBC cell proliferation was associated with the promotion of NLRP3, Caspase-1 p10, IL-1β expression and pyroptosis, suggesting that GOLPH3 expression could play a role in promoting the further development of GBC by promoting cellular pyroptosis (89). In addition, it was found that the NLRP3 inflammasome could enhance phosphorylation of Akt, ERK1/2, and CREB to promote adenocarcinoma proliferation by activating caspase-1 and producing mature IL-1β and IL18. It is suggested that NLRP3 inflammasome-induced cellular pyroptosis may play a role in promoting adenocarcinoma growth (97).

### 3.6 Pancreatic ductal adenocarcinoma

Pancreatic ductal adenocarcinoma (PDA) is one of the most common and aggressive malignancies worldwide (98). PDA morbidity and mortality currently show a rapid upward trend due to changes in dietary and other lifestyle factors (99). Surgery is the only treatment option for localized PDA (100) but ~80% of patients have inoperable cancers due to metastasis at the usually advanced stage on diagnosis (101). The incidence of complications following radical resection is high (102, 103) but few alternative treatments are effective due to resistance (104). In addition, conventional therapy may suppress immune function and activate inflammation (105–108) in patients who already exhibit an aggressive inflammatory and immunosuppressive state (109). PDA lung metastasis was found to be influenced by the tumor microenvironment (TME) (110) and production of cytokines, chemokines and growth factors by tumor-associated macrophages (TAMs) resulting in an immunosuppressive TME which promoted tumor progression and metastasis (111–113). Upregulation of NLRP3 in PDA macrophages regulated TAM polarization and immunogenic or tolerogenic CD4^+^ T cell differentiation and CD8^+^ T cell activation. CD4^+^Th1 cells mediated a tumor protective effect in a mouse PDA model and were strongly associated with prolonged survival of human PDA patients (114). By contrast, CD4^+^ Th2 cells promoted mouse PDA progression and Th2 cell infiltration was strongly associated with reduced survival of human PDA patients (114–116). CD4^+^CD25^+^Foxp3^+^ regulatory T cells (T reg cells) similarly promoted tumor immune escape and CD4^+^ Th17 cells promoted PDA epithelial cell proliferation (117, 118). Deletion of ASC or caspase-1 or pharmacological NLRP3 inhibition reversed the tolerogenic effects of NLRP3^+/+^ TAMs but did not enhance the immunogenic function of NLRP3^-/-^ TAMs. Thus, NLRP3 promoted PDA development (119).

NLRP3-stimulated IL1-β-production has been shown to influence PDA development and progression in human patients and in a mouse model. Elevated IL-1β has been associated with pancreatitis, a recognized risk factor for PDA (120) and high intratumoral and serum IL-1β has been associated with poorer overall survival and increased chemoresistance in PDA patients (121–123). Adipocyte-secreted IL-1β promoted obesity-induced pancreatic carcinogenesis and drug resistance by recruiting tumor-associated neutrophils in a mouse PDA model (124). IL-1β production by PDA-associated myeloid cells may also support tumor progression by promoting immune tolerance (116, 119). Overall, the heterotypic distribution of IL-1β expression in PDA seems to be involved in disease pathogenesis.

### 3.7 Colorectal cancer

Colorectal cancer (CRC) is common and has high morbidity and mortality (125). Genetic predisposition and recurrent inflammatory bowel disease are two major independent risk factors and 80% of colon cancers show mutation of the tumor suppressor colon adenomatous polyposis coli (APC) gene (47). Genes encoding tumor necrosis factor-alpha-inducible protein 3 (TNFAIP3), NLRP3 and NF-κB are prognostic markers for CRC (126). TNFAIP3 is a CRC tumor suppressor but NLRP3 level is associated with poorer survival in patients with aggressive CRC and chronic intestinal inflammation, such as IBD, is considered a risk factor for the development of colorectal cancer (127). Nlrp3*^-/-^* mice developed atypical hyperplasia and tumor formation due to elevated inflammatory responses and disruption of the intestinal epithelial barrier in response to CRC induction by (AOM)/dextransodium sulfate (DSS) and similar results were observed in ASC and caspase-1 deficient mice. Thus, the NLRP3 inflammasome is considered instrumental in resistance to colitis-associated tumorigenesis (128, 129). Reduction of intestinal IL-18 levels in NLRP3 and caspase-1-deficient mice exposed to AOM/DSS showed that recombinant IL-18 prevented tumor development (129). In addition, AOM/DSS-treated IL18^-/-^ and IL18R1^-/-^ mice were more prone to development of intestinal polyps than wild-type mice (12). Therefore, NLRP3-mediated IL-18 secretion promoted differentiation of intestinal epithelial cells, maintained intestinal epithelial integrity and reduced intestinal epithelial cell proliferation during colitis remission, protecting cells from malignant transformation (130). The NLRP3 inflammasome may also inhibit CRC metastasis and proliferation by enhancing the IL-18-induced activity of NK cells independently of INF-γ, in addition to counteracting enterocolitis-associated intestinal carcinogenesis. NLRP3^-/-^ mice have an increased risk of developing CRC liver metastases (131) and caspase-1-deficient mice exhibited more severe tumorigenesis, decreased STAT1 and IL-1β compared with the NLRP3^-/-^ mouse model (132).

Most studies suggest an inhibitory effect of NLRP3 on CRC. However, DSS-induced colitis was attenuated in Nlrp3^-/-^ mice and ameliorated by caspase-1 inhibition. A local decrease in the proinflammatory cytokines, IL-1β, TNF-α and IFN-γ, may be responsible (13). The cancer-promoting function of NLRP3 is also apparent in the interaction of macrophages with tumor cells which enhances CRC invasion and metastasis (133). The NLRP3 inflammasome has also been reported to be highly expressed in mesenchymal-like colon cancer cells (134). During the epithelial-mesenchymal transition (EMT), tumor necrosis factor-α (TNF-α) and transforming growth factor-β1 (TGF-β1) act on NLRP3, although ASC and cleaved caspase-1 do not seem to be involved despite being upregulated in CRC epithelial cell-lines, HCT116 and HT29, leading to cancer progression. Therefore, a necessary condition for the EMT seems to be the expression, rather than the activation, of the NLRP3 inflammasome (134). Overall, the role of the NLRP3 inflammasome in the occurrence and development of colorectal tumors remains controversial and further studies are needed.

RAS mutations are the most common oncogenic mutations in human cancers. The hallmark KRAS mutated cancers are pancreatic cancer, colorectal cancer, lung adenocarcinoma and urogenital tract cancer (135). KRAS is a commonly mutated oncogene in CRC, occurring in approximately 40% of CRC cases; its mutation leads to constitutive activation of KRAS protein, which acts as a molecular switch to continuously stimulate downstream signaling pathways, including cell proliferation and survival, leading to tumorigenesis (136, 137). It was found that in addition to the direct translational effects generated by RAS/MEK/ERK signaling, the inflammation-related effects of KRAS play an important role in tumorigenesis. KrasG^12D^ oncogene-driven myeloproliferation is dependent on NLRP3 inflammasome activation (138). This work revealed the existence of KRAS/RAC1/ROS/NLRP3/IL-1β pathway in human myeloid leukemia. krasG_12D_-induced NLRP3 activation is dependent on RAC1-mediated accumulation of ROS in myeloid leukemia cells.

## 4 NLRP3 and natural killer cell

NK cells are important immune cells, and once activated, NK cells perform cell lysis functions in different ways. First, they can release lysis granules containing perforin and granzyme (139). Then, death receptors such as Fas ligand (FasL) and tumor necrosis factor-related apoptosis-inducing ligand (TRAIL) are also contributors to NK cell-mediated cytotoxicity (140). In addition, NK cells can recognize and induce antibody encapsulation and lysis of target cells *via* CD16 (FcγRIIIa), a process known as antibody-dependent cytotoxicity (ADCC) (141). In addition to direct cytotoxicity, NK cells can regulate innate and adaptive immunity by secreting a range of cytokines, growth factors and chemokines (142).

### 4.1 Function of NK cells in GI cancers

NK cells can act as effector cells and respond to stimuli within a few hours without pre-immunization. Currently, activation of NK cells through the receptor NKG2D is the most well-defined mechanism in tumor surveillance. The level of NKG2D expression in gastric cancer was positively correlated with clinical survival, and *in vitro* experiments confirmed the cytotoxicity of NK cells on gastric cancer cell lines (143). In human pancreatic cancer, NK cells recognize cancer stem cell (CSCs) markers such as CD133 and CD24 in an NKG2D-dependent manner, which is important for exerting cytotoxic (144). NK cells can induce significant apoptosis in the HCC cell line Hep3B through TRAIL/TRAIL (tumor necrosis factor-related apoptosis-inducing ligand) receptor interactions (145). The same mechanism is involved in the NK cell-mediated inhibition of liver metastasis from CRC (146).

In addition to this, NK cells can play an adjuvant function in GI cancers. NK cells can act on other immune cells, such as dendritic cells (DCs) (147), neutrophils (148), and T cells (149), by secreting cytokines, resulting in adaptive immunity.

### 4.2 Role of NLRP3 on NK cells

Tumor cells, stromal cells and other types of immune cells in the TME can influence the function of NK cells. ATP has a key role in the energy metabolism of TME components and influences cancer immunosurveillance. Increased levels of extracellular ATP (eATP) trigger activation of the P2X7-NLRP3-inflammasome, which drives macrophage pyroptosis, enhances maturation and antigen-presentation of dendritic cells (DCs) and improves cytotoxic function of NK cells (150). In a mouse CRC-liver metastasis model, the NLRP3 inflammasome increases IL-18 secretion, promotes maturation of hepatic NK cells, increases FasL expression, and Fas/FasL interaction can exert cytotoxicity on tumor cells (131). The deletion of NLRP3 in human hepatocellular carcinoma can cause upregulation of MICA/B expression which interacts with the NKG2D receptor in NK-92 cells, resulting in cytotoxicity of NK cells (151). Activation of the NLRP3 inflammasome can cause elevated IL-1β levels in TME. *In vivo*, tumor-associated NLRP3/IL-1 signaling induces the expansion of myeloid-derived suppressor cells (MDSCs), leading to reduced NK cell activity (152).

## 5 Therapeutic targeting of NLRP3

The clinical relevance of the NLRP3 inflammasome in GI cancers allows it to be an important molecular target. An important future study is the understanding of the molecular mechanisms of NLRP3 inflammasome activation and the identification of effective NLRP3 inhibitors or inhibitory pathways. Here, we list some inhibitors of NLRP3 that can influence cancer progression (**Table 1**).

**Table 1:** List of NLRP3 inhibitors.

Inhibitor	Mechanism	Effective GI Cancer Type	References
MCC950	Binds Walker B motif, NLRP3 NACHT, ATPase inhibitor	DSS-induced experimental colitis in mice	(153–157)
Compound 6	Binds NLRP3 NACHT domain, blocking ATPase activity and ASC oligomerization		(158)
Fc11a-2	Targets NLRP3 inflammasome, inhibits cytokines release		(159)
VI-16	Inhibits the binding of TXNIP to NLRP3 by reducing NLRP3 activation		(160)
Fraxinellone	Inhibits NF-kappaB pathway and NLRP3 inflammasome activation		(161)
Alpinetin			(162)
Celastrol			(163)
C1-27	Inhibits NLRP3 activation by reducing ASC speck formation	CRC	(164)
Oridonin	Binds NLRP3 NACHT domain, blocks NEK7-NLRP3 interaction	ESCC	(165)
Thalidomide	inhibits caspase-1 activation	/	(166–168)
CY-09	Binds ATP-binding motif, NLRP3 NACHT, inhibits ATPase		(169)
Tranilast	Binds NACHT Inhibits the NLRP3-NLRP3 interaction		(170)
SI-2	Disrupts the interaction between NLRP3		(171)
Canakinumab	IL-1β inhibitor	CRC	(172, 173)
Rilonacept	Binds IL-1β and IL-1α	/	(174)
Anakinra	IL-1 receptor antagonist	CRC	(175)

The diarylsulfonylurea compound MCC950 (originally reported as CRID3/CP-456773) is the most potent and specific NLRP3 inhibitor (153). Mechanistically, MCC950 interacts directly with the Walker B motif within the NLRP3 NACHT structural domain, thereby blocking ATP hydrolysis (154) and inducing the transition of NLRP3 to an inactive conformation (155). In cancer therapy, MCC950 improves T-cell function by reducing the number of immunosuppressive cells, thereby inhibiting and retarding tumor growth (156). In addition, MCC950 was shown to effectively inhibit IL-1β secretion and Caspase-1 activation in ulcerative colitis in mice (157).

A variety of compounds have been shown to be effective in the treatment of experimental colitis in mice caused by DSS. Compound 6, a novel tetrahydroquinoline NLRP3 inhibitor. Specifically inhibits NLRP3 activation *in vivo* by binding to the NLRP3-NACHT structural domain, inhibiting its ATPase activity and blocking ASC oligomerization (158). Fc11a-2, a synthetic small molecule compound that inhibits cytokine release by targeting the NLRP3 inflammasome (159). VI-16 is a ynthetic flavonoid compound that reduces ROS production and inhibits NLRP3 inflammasome activation by inhibiting the binding of TXNIP to NLRP3 (160). Fraxinellone (lactone compound) (161), alpinetin (novel plant flavonoid) (162) and Celastrol (natural triterpene) (163) both are able to affect NLRP3 inflammasome activation by inhibiting NF-kappaB signaling.

NEK7 is a member of the family of NIMA-related kinases (NEKs) and acts as an NLRP3-binding protein capable of regulating their oligomerization and activation. The omega class glutathione transferase (GSTO1-1) inhibitor C1-27 promotes NEK7 deglutathionylation to regulate the release of IL-1β and IL-18 (164), thereby promoting CRC formation. Oridonin, an ent-kaurane diterpenoid, inhibits NLRP3 activation by binding to NLRP3-NACHT and blocking the interaction of NLRP3 with NEK7 (165). Thalidomide, a potent anti-inflammatory agent that inhibits caspase-1 activation (166), has antitumor activity in the treatment of MM and PCa (167, 168). C172 is an inhibitor of the cystic fibrosis transmembrane conductance regulator channel (CFTR), and its analogue CY-09 inhibits the ATPase activity and oligomerization of NLRP3 by binding to its ATP-bound Walker A motif (169). Tranilast is a tryptophan metabolite analogue that blocks NLRP3-NLRP3 interactions and oligomers by binding to the NACHT structural domain of NLRP3, but does not affect its ATPase activity (170). SI-2, an acetylase inhibitor, specifically inhibits NLRP3 inflammasome activation by disrupting the interaction between NLRP3 and ASC and blocking ASC spot formation (171).

Although small molecules and drugs have been shown to modulate inflammasome activity, IL-1 signaling blockade is currently being used clinically to treat NLRP3-driven immune disorders. Three biologics are approved by the U.S. Food and Drug Administration for the treatment of multiple inflammatory diseases: Canakinumab, a human anti-IL-1β monoclonal antibody, has significant antitumor effects in NSCLC (172, 173); Rilonacept, a decoy receptor that binds IL-1β and IL-1α (176); Anakinra is a recombinant IL-1 receptor antagonist (IL-1RA) that blocks IL-1α and IL-1β signaling *via* IL-1R (174). In addition, the combination of Anakinra with 5-FU and bevacizumab improved the survival and overall survival of patients affected by CRC (175).

In summary, targeting the NLRP3 inflammasome or its downstream pathways as a research target has begun to attract attention as a potential strategy for the development of novel anticancer therapies.

## 6 Conclusions

Evidence suggests that NLRP3 plays a dual role in tumorigenesis and anticancer immunity (**Table 2**). However, its roles in different tumor types, at different stages of tumor development and mechanisms of action in tumor formation, development and invasion all remain controversial. Pyroptosis may induce varied outcomes in different tumors, meriting unprecedented attention in the field of tumor therapy. Inhibition or promotion of pyroptosis is a new approach to tumor therapy. However, pathways of pyroptosis in tumors and molecular mechanisms still lack convincing explanations. The NLRP3 inflammasome mediates pyroptosis and a study of its *in vivo* impact on the tumor process may allow applications of PCD targeting in tumor therapy.

**Table 2:** Role of NLRP3 inflammasome activation or suppression in cancer development.

Type of cancer	Source of experimental evidence	Effect on tumor	References
OSCC	-OSCC and adjacent normal tissue NLRP3^-/-^ mouse	promoting effect	(42–44, 46)
ESCC	-ESCC and adjacent normal tissue ESCC cell lines Het-1A	promoting effect	(51, 52)
GC	-GC tissue -GC cell lines (SGC-7901, BGC-823, HGC-27 and AGS) -normal gastric epithelial cell line (GES-1)	Tumor promoting effect	(11, 19, 59, 63)
HCC	-HCC and adjacent normal tissue	suppressor effect	(66–70, 81)
GBC	-GBC and adjacent normal tissue	promoting effect	(85, 89–97)
PDA	ASC^-/-^ mouse Caspase-1^-/-^ mouse NLRP3^-/-^ mouse -PDA and adjacent normal tissue	promoting effect	(116, 119)
CRC	-CRC and adjacent normal tissue NLRP3^-/-^ mouse IL-18^-/-^ mouse IL18R1^-/-^ mouse Colon cancer epithelial cells HCT116 and HT29	promoting effect suppressor effect	(12, 128–131, 133, 134).

## Author contributions

YW designed the review article. JW wrote the manuscript and prepared the figures and tables. BX, YL, LW, LH, XM and TZ revised the manuscript. All authors contributed to the article and approved the submitted version.
